# Supplementary material for: Histone Deacetylase Inhibitors and Microtubule Inhibitors Induce Apoptosis in Feline Luminal Mammary Carcinoma Cells
Source: Animals (Basel). 2021 Feb 15;11(2):502. doi: 10.3390/ani11020502 (PMC7918990; doi:10.3390/ani11020502)
Supplement: Supplementary file 1 [file animals-11-00502-s001.pdf]

Article

Histone Deacetylase Inhibitors and Microtubule Inhibitors Induce Apoptosis in Feline Luminal Mammary Carcinoma Cells

Filipe Almeida [1,2], Andreia Gameiro [1], Jorge Correia [1] and Fernando Ferreira [1,*]

[1] CIISA - Centro de Investigação Interdisciplinar em Sanidade Animal, Faculdade de Medicina Veterinária, Universidade de Lisboa, Avenida da Universidade Técnica, 1300-477 Lisboa, Portugal; filipe.almeida@insa.min-saude.pt (F.A.); agameiro@fmv.ulisboa.pt (A.G.); jcorreia@fmv.ulisboa.pt (J.C.)
[2] Antiviral Resistance Laboratory, Infectious Diseases Department, National Institute of Health Dr. Ricardo Jorge, Av. Padre Cruz, 1649-016, Lisbon, Portugal
* Correspondence: Correspondence: fernandof@fmv.ulisboa.pt; Tel.: +351-21-365-2800 (Ext. 431234)

Citation: Almeida, F.; Gameiro, A.; Correia, J.; Ferreira, F. Histone Deacetylase Inhibitors and Microtubule Inhibitors Induce Apoptosis in Feline Luminal Mammary Carcinoma Cells . *Animals* **2021**, *11*, 502. https://doi.org/10.3390/ ani11020502

Academic Editor: Mandy Paterson
Received: 8 December 2020
Accepted: 12 February 2021
Published: 15 February 2021

Publisher's Note: MDPI stays neutral with regard to jurisdictional claims in published maps and institutional affiliations.

Copyright: © 2021 by the author. Licensee MDPI, Basel, Switzerland. This article is an open access article distributed under the terms and conditions of the Creative Commons Attribution (CC BY) license (http://creativecommons.org/licenses/by/4.0/).

Supplementary

Table S1. Evaluation criteria for ER and PR staining.

ER / PR			
**% of positive staining**		**Average staining intensity**	
**Score**	**Interpretation**	**Score**	**Interpretation**
0	No signal	0	No signal
1	<1%	1	Weak
2	1 – 10%		
3	10 – 33%	2	Average
4	33 – 66%	3	Strong
5	>66%		
**Score**	**0 - 8**	Positive staining (0-5) + Average staining intensity (0-3)	

Table S2. Evaluation criteria for Ck 5/6 and Ki-67 staining.

**Ck 5/6 scoring criteria**		**Ki-67 scoring criteria**	
**%**	**Interpretation**	**%**	**Interpretation**
<1%	Negative	<14%	Weak
≥1%	Positive	≥14%	Strong

Table S3. Evaluation criteria for HER2 staining.

**HER2 scoring criteria**	
**Score**	**Interpretation**
0	No signal
+1	Weak, incomplete membrane staining
+2	Weak or non-uniform, complete membrane staining and obvious distribution in at least 10% of cells
+3	Intense and uniform staining of at least 10% of invasive tumor cells
